# Plant Secondary Metabolites on Efflux-Mediated Antibiotic Resistant *Stenotrophomonas Maltophilia*: Potential of Herbal-Derived Efflux Pump Inhibitors

**DOI:** 10.3390/antibiotics12020421

**Published:** 2023-02-20

**Authors:** Thi Huyen Thu Nguyen, Ngoc Anh Thơ Nguyen, Hai Dang Nguyen, Thi Thu Hien Nguyen, Mai Huong Le, Minh Quan Pham, Huu Nghi Do, Kim Chi Hoang, Serge Michalet, Marie-Geneviève Dijoux-Franca, Hoang Nam Pham

**Affiliations:** 1 Doctoral School, University of Science and Technology of Hanoi, Vietnam Academy of Science and Technology, 18-Hoang Quoc Viet, Cau Giay, Hanoi 10072, Vietnam; 2 Saint Paul Hospital, 12 Chu Van An, Ba Dinh, Hanoi 11114, Vietnam; 3 UMR 5557, Ecologie Microbienne, CNRS, INRAe, VetagroSup, UCBL, Université de Lyon, 43 Boulevard du 11 Novembre, F-69622 Villeurbanne, France; 4 Department of Academic Affairs, University of Science and Technology of Hanoi, Vietnam Academy of Science and Technology, 18-Hoang Quoc Viet, Cau Giay, Hanoi 10072, Vietnam; 5 Institute of Biological and Food Technology, Hanoi Open University, B101 Nguyen Hien, Hai Ba Trung, Hanoi 11615, Vietnam; 6 Institute of Natural Products Chemistry, 1H building, Vietnam Academy of Science and Technology, 18-Hoang Quoc Viet, Cau Giay, Hanoi 10072, Vietnam; 7 Department of Biotechnology, Graduate University of Science and Technology, Vietnam Academy of Science and Technology, 18-Hoang Quoc Viet, Cau Giay, Hanoi 10072, Vietnam; 8 Department of Life Sciences, University of Science and Technology of Hanoi, Vietnam Academy of Science and Technology, 18-Hoang Quoc Viet, Cau Giay, Hanoi 10072, Vietnam

**Keywords:** efflux pump inhibitor, plant metabolites, antimicrobial activity, bacterial multidrug resistance, metal contamination

## Abstract

During the process of adapting to metal contamination, plants produce secondary metabolites that have the potential to modulate multidrug-resistant (MDR) phenotypes; this is achieved by inhibiting the activity of efflux pumps to reduce the minimum inhibitory concentrations (MICs) of antimicrobial substrates. Our study evaluated the effect of secondary metabolites of belowground parts of *Pteris vittata* L. and *Fallopia japonica,* two metal-tolerant plants from northern Vietnam, on six antibiotic-resistant *Stenotrophomonas maltophilia* strains possessing efflux pump resistance mechanisms that were isolated from soil and clinical samples. The chemical composition of aqueous and dichloromethane (DCM) fractions extracted from *P. vittata* and *F. japonica* was determined using UHPLC-DAD-ESI/QTOF analysis. The antibacterial and efflux pump inhibitory activities of the four fractions were evaluated for the six strains (K279a, 0366, BurA1, BurE1, PierC1, and 502) using a microdilution assay at fraction concentrations of 62.5, 125, and 250 μg/mL. The DCM fraction of *F. japonica* exhibited remarkable antibacterial activity against strain 0366, with a MIC of 31.25 μg/mL. Furthermore, this fraction also significantly decreased gentamicin MIC: four-fold and eight-fold reductions for BurA1 and BurE1 strains, respectively (when tested at 250 μg/mL), and two-fold and eight-fold reductions for K279a and BurE1 strains, respectively (when tested at 125 μg/mL). Pure emodin, the main component identified in the DCM fraction of *F. japonica*, and sennidine A&B only reduced by half the MIC of gentamicin (when tested at 30 μg/mL). Our results suggest that the DCM fraction components of *F. japonica* underground parts may be potential candidates for new bacterial efflux pump inhibitors (EPIs).

## 1. Introduction

*Stenotrophomonas maltophilia* is a Gram-negative bacterium found in the environment and considered an opportunistic pathogen. Its various strains are found both in natural and anthropogenic environments, such as soil contaminated with heavy metals, causing numerous infections in hospitals [1]. Clinical *S. maltophilia* strains possess a high capacity for resistance against different antibiotics, including aminoglycosides, beta-lactam, carbapenem, quinolones, and polymixines [2]. Moreover, several studies have shown that clinical and environmental isolates of *S. maltophilia* are multidrug-resistant (MDR), and phenotypically and taxonomically homogeneous [1]. 

*S. maltophilia* has developed multiple resistance mechanisms resulting in its intrinsically low sensitivity to antibiotics, to which multidrug efflux pumps contribute significantly causing its MDR phenotype [2]. These pumps bind various substrates, including most antibiotics (ATB), leading to reduced intracellular ATB levels, and thus to inactivity and resistance [3]. The overexpression of multidrug efflux pumps is associated with clinical drug resistance. Furthermore, the function of efflux pumps is not only restricted to antibiotic resistance but it is also involved in plant–bacteria interactions, for example, in the detoxification of both defense metabolites and toxic factors such as plant antimicrobials and heavy metals [2,3]. Therefore, inhibition of the multidrug efflux pump, as an adjuvant combination therapy with other small molecules that block efflux pump activities, is a promising approach to restore the efficacy of existing antibiotics. This would enhance the activity of antibiotics and reduce the emergence of MDR bacteria [4]. Furthermore, plants have developed two main adaptive strategies that help them to cope with an excess of heavy metals: (i) exclusion, which is the most common strategy, is where metal concentration in the shoots remains low and constant while the external concentration constantly varies, and (ii) accumulation occurs, where metal is absorbed and translocated to the aboveground plant biomass, which can then be harvested and removed easily [5,6]. 

*Fallopia japonica* (Houtt.) Ronse Decr *(syn. Reynoutria japonica* Houtt*.; Polygonum cuspidatum* Siebold & Zucc.), also called Japanese knotweed, is known as a plant able to grow in metal-polluted soil. The proportion of dihydroanthracenone derivates found in *F. japonica* root extract, including torosachrysone, anthraquinone, and dianthrone, are increased by metal contamination [5]. Recent research from 2020 reported a correlation between the abundance of rhizospheric *Stenotrophomonas* and the concentration of emodin, a bioactive compound identified in root extract of *F. japonica*. This compound protects plants against toxic factors but also possess an antimicrobial effect. The research showed that the combined impact of emodin could assist *F. japonica* growth under the dominance of *Stenotrophomonas* in the root microbiome [7].

*Pteris vittata* L., commonly found in tropical and subtropical areas such as Southeast Asia, Africa, and Australia, has been reported to be an excellent metal hyperaccumulator. In 2001, Ma et al. proposed that *P. vittata* in Florida could hyperaccumulate up to 22,630 mg/kg in leaves [8]. Moreover, *P. vittata* could grow and develop normally on Zn-contaminated soil with the ability to accumulate Zn up to 737 mg/kg in the leaves [9]. Therefore, the phytoremediation capability and the molecular mechanism of metal tolerance in this fern have been the focus of many studies [9,10,11,12]. In Vietnam, to remediate metal-contaminated soil in Thai Nguyen using plants, *P. vittata* and some other ferns were employed; the concentrations of As that accumulated in the roots and leaves reached 6042 ppm and 3756 ppm. As a result, they could tolerate soils highly contaminated with 15,146 ppm As, 5000 ppm Pb, and 1200 ppm Cd [11]. In addition, the mechanism of metal hyperaccumulation in *P. vittata* dominating Thai Nguyen mining sites has been revealed using a combination of ionomics and metabolomics analysis [13]. In the pharmacological field, some studies have shown that *P*. *vittata* possesses antioxidant, hypoglycemic, anti-inflammatory, antiplatelet aggregation, and antitumor activities [14,15,16]. Recently, a total of 139 secondary metabolites, including seventy flavonoids, sixteen phenylpropanoic acid derivatives, five coumarins, two stilbenoids, fourteen benzoic acids, nine phenols, twenty alkaloids, and three terpenoids, were found in the *P. vittata* metabolome [14]. Among them, quercetin showed remarkable properties as an antibacterial agent and an efflux pump inhibitor (EPI). When administered together with other compounds, it increased the subcutaneous bioavailability of moxidectin in lambs [17], the subcutaneous bioavailability of paclitaxel in rats [18], and the oral bioavailability of digoxin in pigs [19]. The antimicrobial activity of other compounds, such as luteolin, kaempferol, and apigenin, was also reported [20]. Additionally, Pb and Zn contamination resulted in an elevated abundance of genera belonging to MDR opportunistic pathogens known to harbor different efflux pumps such as *Burkholderia* spp., *Acinetobacter* spp., or *Cupriavidus* spp. in the rhizosphere of *P. vittata* [6]. 

The present study evaluates the antibacterial effect and the efflux pump inhibitory activities of secondary metabolites extracted from the aqueous and dichloromethane (DCM) fractions of belowground parts of *P. vittata* and *F. japonica* on six *S. maltophilia* strains isolated from soils and clinical samples. 

## 2. Results

In this study, the chemical composition, minimum inhibition concentration (MIC), and efflux pump inhibitory activities of *P. vittata* and *F. japonica* extracts against six strains of *S. maltophilia* that potentially expressed efflux pumps were determined. 

### 2.1. Chemical Composition of Fractions

**Figure A1:**
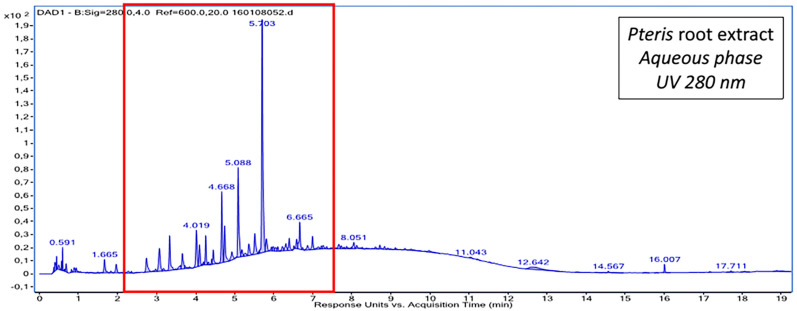
Ultra high performance liquid chromatography (UHPLC) chromatogram with UV detector at 280 nm of *Pteris vittata* L. roots aqueous fraction.

**Figure A2:**
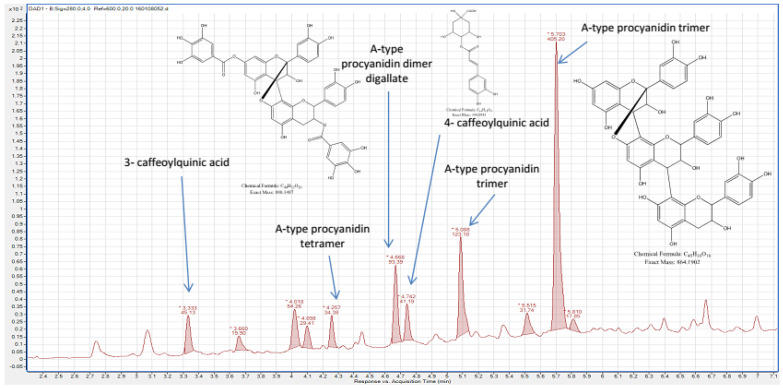
Chemical composition of *P. vittata* L. roots aqueous fraction from the tape-strips analysis.

**Figure A3:**
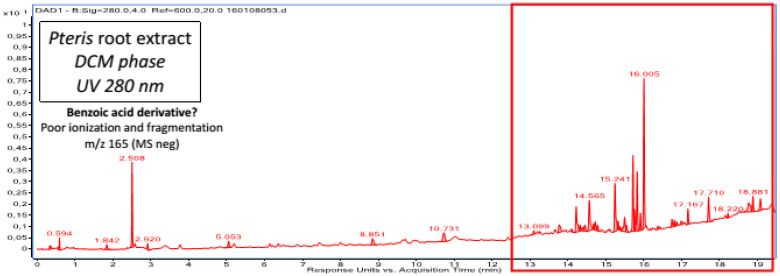
UHPLC chromatogram with UV detector at 280 nm of *P. vittata* L. roots dicholoromethane (DCM) fraction.

**Figure A4:**
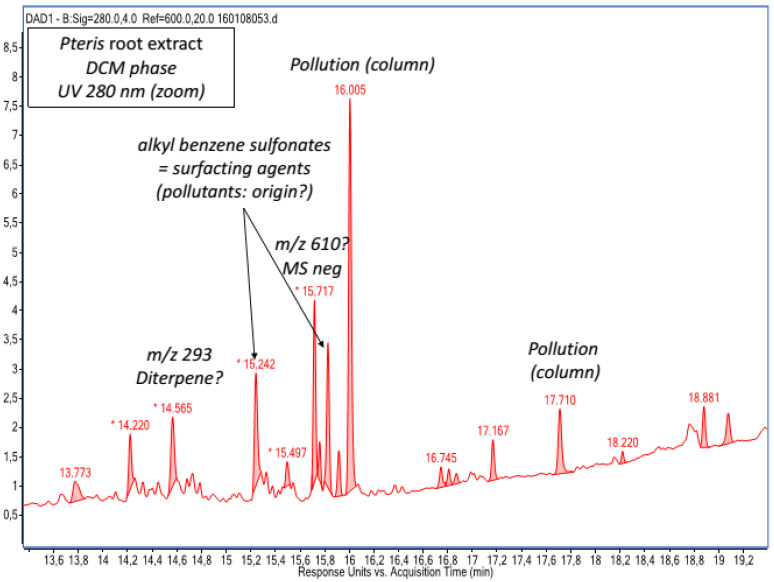
Chemical composition of *P. vittata* L. roots DCM fraction from the tape-strips analysis.

**Figure A5:**
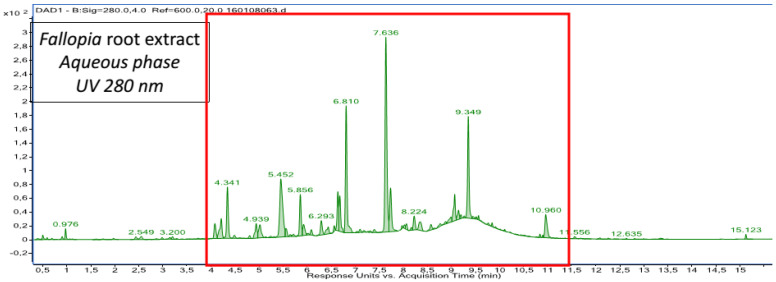
UHPLC chromatogram with UV detector at 280 nm of *F. japonica* roots aqueous fraction.

**Figure A6:**
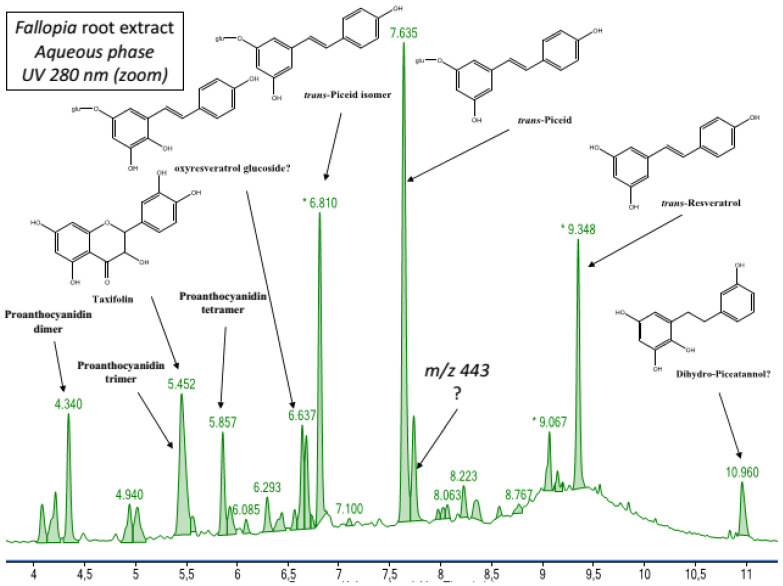
Chemical composition of *F. japonica* roots aqueous fraction from the tape-strips analysis.

**Figure A7:**
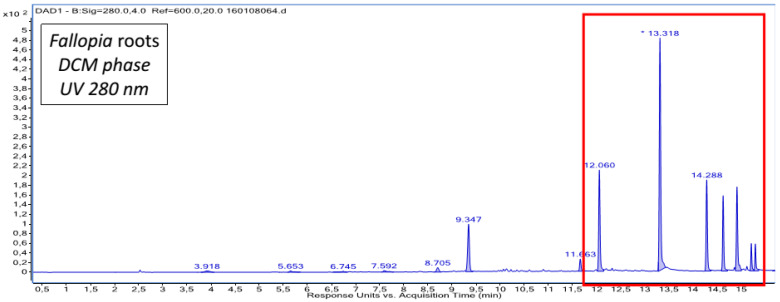
UHPLC chromatogram with UV detector at 280 nm of *F. japonica* roots DCM fraction.

**Figure A8:**
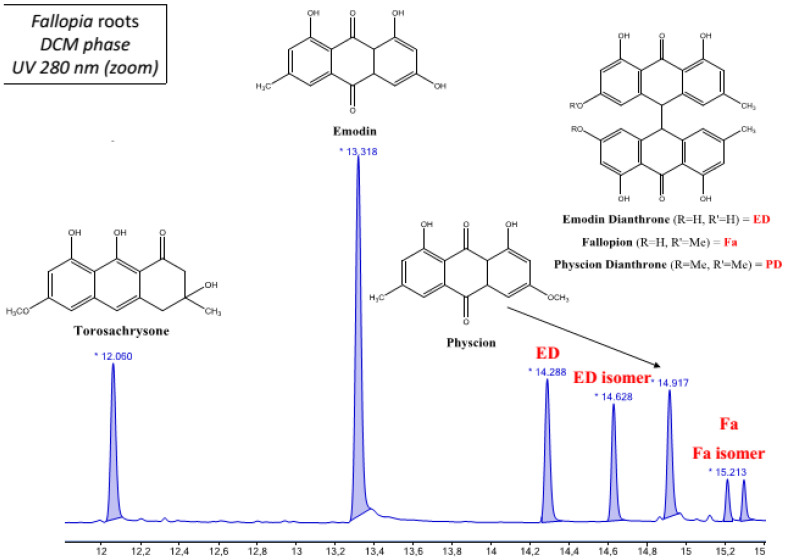
Chemical composition of *F. japonica* roots DCM fraction from the tape-strips analysis.

The main components of the aqueous fractions of *P.vittata* and *F. japonica* were A-type polymeric procyanidins together with caffeoyl-quinic acid derivatives, and *trans*-reseveratrol derivatives together with B-type polymeric procyanidins, respectively. In the *P. vittata* DCM fraction, an unidentified diterpene was the only compound tentatively annotated, whereas in the *F. japonica* DCM fraction, emodin, emodin dianthrone isomers, physcion, torosachrysone, and fallopion isomers were the major secondary metabolites detected. The chemical composition of the four fractions is shown in Table 1 and Figure A1, Figure A2, Figure A3, Figure A4, Figure A5, Figure A6, Figure A7 and Figure A8 (Appendix A).

### 2.2. Minimum Inhibitory Concentration Determination

The MIC is defined as the concentration at which no visible bacterial growth is observed. The MICs found for all extracts were above 2000 μg/mL, except for the DCM fraction of *F. japonica* which exhibited noteworthy antibacterial activity against strain 0366 with a MIC of 31.25 μg/mL (Table 2). 

### 2.3. Efflux Pump Inhibitory Activity Evaluation

First, we examined the six *S. maltophilia* strains for their ability to resist ATB by potentially using efflux pump mechanisms. For that, gentamycin MIC was evaluated on the six *S. maltophilia* strains in the presence or absence of phenylalanine-arginine-β-naphthylamide (PAβN) at 25 μg/mL, a commercial EPI. For strains K279a, BurA1, and BurE1 a reduction of gentamycin MIC was observed (with up to four-fold reduction for BurE1), whereas, for the three other strains (502, 0366, PierC1), PAβN did not reduce gentamicin MIC (Table 3). This let us hypothesize that efflux is not a mechanism of resistance mainly expressed and used to resist gentamycin in these strains (which are also notably more sensitive to gentamycin), so the efflux pump inhibitory activity assays were not conducted on these strains. 

The highest concentration chosen for the efflux pump inhibitory activity assay was 250 μg/mL, which is more than four-fold lower than the lowest MIC of 1000 μg/mL (Table 2). The MIC of gentamicin was remarkably reduced by four-fold and eight-fold with the *F. japonica* DCM fraction for BurA1 and BurE1, respectively. At 125 μg/mL, a two-fold reduction was observed for K279a and BurA1, and a four-fold reduction for BurE1 (Table 4). At 62.5 μg/mL, the gentamicin MIC was decreased by two times for K2798a and BurE1. For BurE1 and BurA1, a linear relationship between fraction concentration and gentamycin MIC was observed, whereas this was not the case for K279a.

**Figure A9:**
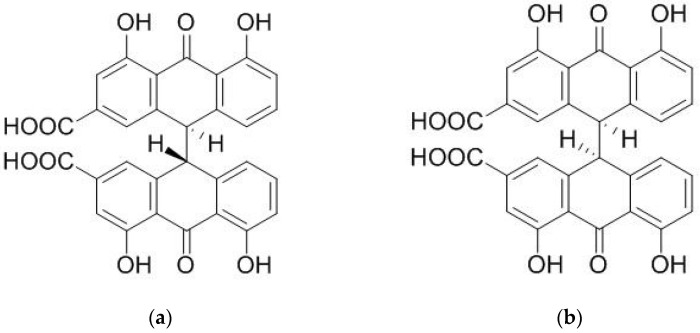
Chemical structure of (**a**) sennidine A and (**b**) sennidine B.

To evaluate the inhibitory activity of the main components of the *F. japonica* DCM fraction, we conducted the efflux pump inhibitory activity assay on commercially pure emodin, physcion, sennidine A, and sennidine B. Sennidine A and sennidine B were chosen because they possess the same dianthrone structure as emodin dianthrone or fallopion, two main secondary metabolites detected in *F. japonica* DCM fraction but which are not commercially available (Figure A9). The concentration used was 30 μg/mL. Emodin, sennidine A, and sennidine B reduced gentamicin MIC by half for K279a and BurE1 strains, while physcion had no effect (data not shown). In addition, all four compounds showed very poor antibacterial activity (less than 10%) on the growth of the six strains (Table 5).

## 3. Discussion

*F. japonica* belowground parts contain bioactive compounds such as anthraquinones, stilbene, and procyanidin derivatives, which possess known pharmacological effects [21,22,23]. We detected and identified mainly anthraquinones (monomers: emodin, physcion; and dimers: emodin dianthrone and fallopion) in the *F. japonica* DCM fraction exhibiting efflux pump inhibitory activity. Polydatin and revesratrol derivatives were also found in this fraction but in lower abundance. The *F. japonica* DCM fraction also exhibited moderate antibacterial activity on the *S. maltophilia* strains tested (except for strain 0366, for which significant antibacterial activity was observed). Previous studies reported high antibacterial efficacy of anthraquinones against Gram-positive bacteria such as *Bacillus cereus*, *Listeria monocytogenes*, *Staphylococcus aureus ATCC25922* or *Salmonella anatum* [24]. Another in vitro study revealed noticeable inhibitory effects against the Gram-negative *Haemophilus parasuis* of emodin extracted from *F. japonica* [25]. However, only the DCM fraction of *F. japonica* showed a moderate antibacterial effect when tested with *S. maltophilia*. This is not surprising considering that *S. maltophilia* strains are known to exhibit elevated multidrug resistance by forming a natural protective layer against the host immune defense system and several antimicrobial agents using intrinsic or acquired resistance mechanisms [26,27]. For the *S. maltophilia* 0366, our unpublished data showed that this clinical strain was resistant to antibiotics of the β-lactam (ticarcillin, piperacillin, imipenem) and quinolone groups (ciprofloxacin and pefloxacin), but it was sensitive to a combination of a β-lactam and a β-lactamase inhibitor (ticarcillin + clavulanic acid), aminoglycosides group (gentamicin, isepamicin, tobramycin), polymyxin (colistin) and trimethoprim/sulfamethoxazole. In our study, three *S. maltophilia* strains (K279a, BurA1, and BurE1) previously showed in vitro resistance to almost all the antibiotic classes including aminoglycosides, penicillin, carbapenems, polymyxin, and monobactam [2]. The difference in antimicrobial resistant profile between the *S. maltophilia* 0366 and other *S. maltophilia* strains may be one of the reasons justifying the low MIC (31.25 μg/mL) obtained for *F. japonica* DCM fraction against this strain. We suggest that the *F. japonica* DCM fraction could possess several secondary metabolites including anthraquinones and resveratrol derivatives that might be active against *S. maltophilia* 0366. However, the reasons for the sensitivity of this strain remain to be elucidated. Therefore, it is envisaged to isolate the compounds from the *F. japonica* DCM fraction and evaluate their antibacterial activity against the *S. maltophilia* 0366 strain in our future works.

The importance of efflux pumps and their role in antibiotic resistance is increasing. As a result, the inhibition of multidrug efflux pumps is a promising approach to restore the efficacy of existing antibiotics as adjunctive therapies and thus contribute to the reduction of the emergence of MDR bacteria [4]. In this study, PaβN, an EPI used in clinical settings, was used as a positive control for efflux inhibition [28]. Different strategies to avoid efflux pump-driven resistance have been proposed, including: (1) the development of new antibiotics unrecognized by efflux pumps, (2) inducing functional change in efflux pumps through structural modifications, (3) the inhibition of the energy sources needed for efflux pumps, (4) inducing a decrease in the expression of efflux pumps genes, and (5) the competitive/non-competitive inhibition of efflux pumps [29,30]. PAβN is a promising inhibitor with an effective mode of efflux pump inhibition, as well as broad host (*P. aeruginosa*, *K. pneumoniae*, *C. jejuni*, *E. coli*, *S. typhimurium*, and *E. aerogenes*) and antibiotic (levofloxacin, oxazolidinones, chloramphenicol, rifampicin, macrolides/ketolides, and gentamicin) ranges [28,31,32]. In our research, PAβN did not reduce the gentamicin’s MIC for three *S. maltophilia* strains (502, 0366 and PierC1), therefore the efflux pump inhibitory activity assay was not performed on these strains. They may employ different mechanisms of resistance than efflux pumps, as reported elsewhere [2]. The efflux pump inhibitory activity test was conducted for three other strains (K279a, BurA1, and BurE1), for which the MIC of gentamicin was reduced in the presence of PaβN (two-fold for K279a and BurA1, and eight-fold for BurE1) (Table 3). Our results showed that the DCM fraction of *F. japonica* with emodin, emodin dianthrone, physcion, and fallopion as its main components was the most active fraction tested. At the maximum concentration tested (250 µg/mL), this extract reduced gentamicin MIC on both clinical and environmental strains (two times for K279a, four times for BurA1 and eight times for BurE1) (Table 4). Emodin was also reported as a strong inhibitor of P-glycoprotein in the MDR1-transfected Madin–Darby Canine Kidney II (MDR1-MDCK II) and Caco-2 cells (IC_50_=9.42 μM) [29], and inhibited the expression of the ATP-binding cassette super-family G member (ABCG2) in gallbladder carcinoma [30]. Specifically, this compound was able to reduce both amoxicillin MIC and mRNA expression of *hefA*, a gene encoding a member of the active efflux system in *Helicobater pylori* [33]. A recent study hypothesized that emodin could promote the recruitment of *Stenotrophomonas* in *F. japonica*’s root microbiome either directly, by promoting the growth of *Stenotrophomonas*, or indirectly, by inhibiting the growth of other microorganisms to assist *F. japonica* growth [7]. Moreover, the proportions of anthraquinone derivatives (mainly torosachrysone and dianthrones) from the root extract of *F. japonica* were found to be raised under metal stress [5]. Additionally, resveratrol has been reported as an inhibitor of the ethidium bromide efflux pump in *Mycobacterium smegmatis* [34].

Regarding efflux pump inhibitory activity, the DCM fraction of *P. vittata* L did not significantly reduce gentamicin’s MIC. However, we were able to identify diterpenes: a group of terpenoids from the DCM fraction of *P. vittata*. Several terpenoids were reported to exhibit a broad spectrum of antibacterial activity [35]. In traditional medicine, *P. vittata* is commonly used in the treatment of dysentery and diarrhea caused by bacterial infections [19,36]. In different solvents (MeOH, water, acetone, and petroleum ether), the *P. vittata* extract inhibited *E. coli, B. subtilis, P. aeruginosa, S. aureus,* and *Proteus vulgaris* at different concentration levels [37]. In our results, the *P. vittata* fractions did not show significant antibacterial and efflux pump inhibitory activity. Therefore, optimizing the *P. vittata* extraction process will be necessary for future research. 

*S. maltophilia,* commonly found in the environment, is an opportunistic bacterial pathogen that is resistant to common antibiotics and causes several infections in hospitals [1,38]. The first choice of treatment is usually trimethoprim-sulfamethoxazole [38]. However, numerous studies have reported that the proportion of strains that are susceptible to trimethoprim-sulfamethoxazole has been declining, with only 38,7% showing resistance due to an increase in intrinsic resistance [1,38]. The major intrinsic resistance mechanism responsible for the MDR phenotype could be attributed to the activity of chromosomally encoded multidrug efflux pumps [2,39,40]. A clinical isolate of an *S. maltophilia* strain with high MIC to several antibiotics was found to coordinately hyper-express the Resistance-Nodulation-Division (RND) family efflux pumps SmeZ and SmeJK [41,42]. MDR pumps are not only limited in the clinical environment but also play a role in natural ecosystems such as soil and the rhizosphere, with cross-interactions between plants, soil and bacteria. For example, the elevated expression of the SmDEF pump, which is responsible for the resistance to quinolone in *S. maltophilia,* is the result of the flavonoid binding to the transcriptional repressor *SmeT* [43]. Additionally, environmental factors such as oxidative stress, metal contamination, or antibiotics may also cause the overexpression of efflux pumps. In our research, we studied efflux pump inhibitory activity toward three *S. maltophilia* strains, including K279a, BurA1, and BurE1 which have previously shown in vitro resistance to almost all the antibiotic classes including aminoglycosides, penicillins, carbapenems, polymyxin and monobactam [2]. The RND efflux pumps SmeDEF, SmeGH, SmeIJK, SmeMN, SmeOP-TolC, SmeVWX, and SmeYZ were also found in all three strains and are involved in their MDR properties [2]. It has been reported that the SmIJK, SmeYZ, and SmeOP*-TolC* pumps played an important role in the extrusion of aminoglycosides in *S. maltophilia* [44,45,46]. Furthermore, the genes encoding the ABC transporter protein and the MFP that are homologous to *macA* and *macB* were identified in all three strains [2]. It has been proven, according to the literature, that the MacABCsm efflux pumps provide intrinsic resistance in *S. maltophilia* to aminoglycosides [47]. Therefore, it would be interesting and necessary to determine the expression levels of the efflux pump genes *MacABCsm*, *SmeIJK*, *SmeYZ*, and *SmeOP-TolC* in the *S. maltophilia* BurA1, BurE1, and K279a strains using quantitative real-time reverse transcription PCR. It is also important to compare them to the expression levels of three other tested *S. maltophilia* strains (502, 0366, and PierC1) and the negative control (e.g., obtained through gene deletion approach) to confirm the critical role of these efflux pump genes in antibiotic resistance contributing to MDR phenotypes in these strains. Most importantly, the main components of the DCM fraction will be purified to evaluate their individual antibacterial and efflux pump inhibitory activities.

## 4. Materials and Methods

**Plant materials**

*P. vittata* L. and *F. japonica* belowground parts were collected in Dai Tu, Thai Nguyen, and were identified by Dr. Do Van Hai at the Institute of Biological Resources, Vietnam Academy of Science and Technology. The voucher of specimens was deposited in the Laboratory of Life Sciences Department, Hanoi University of Science and Technology, Vietnam Academy of Science and Technology.

**Bacterial and growth conditions**

The tested bacteria were various *Stenotrophomonas maltophilia* strains (such as 502, 0366, K279A, PierC1, BurA1, and BurE1). Among them, 502, 0366, and K279a were obtained from clinical samples [25] while the remaining strains were isolated from soils in France (PierC1) and Burkina Faso (BurA1 and BurE1) [26]. These strains differ in their antibiotic resistance profiles.

Bacterial strains were grown in nutrient agar medium (BD Co., Franklin Lakes, NJ, USA), and incubated at 28 °C for 24 h. A concentration of 0.8 McFarland (10^8^ CFU/mL) in sodium chloride 0.9% of each strain was obtained using a densimeter (bioMerieux Inc., Durham, NC, USA). These suspensions were then diluted in nutrient broth to obtain stock solutions containing 10^6^ CFU/mL.

**Preparation of plant extracts**

The dried belowground parts of *P. vittata* L. and *F. japonica* were grinded after freeze-drying and extracted three times with solvent mixture of MeOH and distilled water 1:1 (*v*/*v*), then with 100% MeOH (1 mL of solvent for 10 mg of plant material). The two solvent extracts were combined and the MeOH was evaporated under reduced pressure (Büchi 461 Water Bath, Flawill, Switzerland). The extracts were then transferred into a separatory funnel and partitioned against equal volumes of DCM to afford two fractions: the aqueous fraction and the DCM-soluble fraction. All extracts were concentrated until a constant weight was obtained.

**Chemical composition of plant fractions**

The main components of the aqueous and DCM phases for the two tested plants were analyzed using UHPLC-DAD-ESI/QTOF analysis performed on an Agilent Infinity® 1290 system with a UV/vis DAD G4212A detector and an q-TOF 6530 detector controlled using the MassHunter® software (Agilent Technologies®, Santa Clara, CA, USA). The putative annotation of the main compounds in each fraction was based on UV, HRMS, and MS-MS spectra, and relative retention times. Data were compared with previous literature reports for compounds in *Pteris* spp., *Fallopia* spp., or species in other genera.

**Minimum inhibitory concentration (MIC) determination**

The antibacterial activities of both aqueous and DCM fractions extracted from *P. vittata* L. and *F. japonica* dried roots were evaluated by means of the micro-dilution assay against the six bacterial strains of *S. maltophilia* (502, 0366, K279A, PierC1, BurA1, and BurE1) [39,40]. The stock solutions of all the extracts (80 mg/mL) were prepared in 20% dimethylsulfoxide (DMSO/water). Two-fold serial dilutions were made with 20 % DMSO. Then, these solutions were prepared in nutrient broth in the ratio of 5:95, finally producing concentrations between 31.25 and 4000 μg/mL. A volume of 100 μL of each solution was first introduced to a 96-well microtiter plate (one concentration in one row). Equal volumes (100 μL) of bacterial suspension yielding an approximate inoculum size of 10^6^ colony forming units (CFU)/mL in nutrient broth were added to the wells to obtain final concentrations of each fraction between 15.625 and 2000 μg/mL. The plates were sealed and incubated at 28 °C for 24 h. Bacterial growth was assessed by determining the OD of the solution in each well at 600 nm. The minimum inhibitory concentration (MIC) was determined as the lowest concentration at which the bacterial growth was inhibited by over 90%. Triplicate wells were measured and the data were then averaged. In cases where complete inhibition occurred at the lowest concentration (15.625 μg/mL), further serial dilutions were performed until a MIC endpoint was reached. Sulfamethoxazol (Sigma-Aldrich Inc., St.Louis, MO, USA) (at concentrations of 20 and 40 mg/mL) was used as the positive antibacterial control. The final DMSO concentration in each well was 0.5% per well, and sterile broth was used as negative controls. At this percentage, DMSO had no effect on bacterial growth.

**Efflux pump inhibitory activity evaluation**

The MIC of the tested antibiotic was determined by two-fold serial broth microdilution in 96-well plates in the presence or absence of each extract at the indicated concentration, which was four-fold lower than its MIC as determined in the above-mentioned test. Gentamicin (DuchefaBiochemie B.V., Haarlem, Netherland) was the antimicrobial agent used at concentrations that varied from 0.625 to 80 μg/mL. Each well contained 5 μL of the antibiotic, 5 μL of the extract, 90 μL of the NB medium, and 100 μL of the NB inoculum containing 10^6^ CFU/mL. The microtiter plates were incubated at 28 °C for 24 h before the results were recorded. Bacterial growth was determined by measuring the OD of the solution in each well by using a TECAN Infinite® 200 Pro microplate reader (TECAN Group Ltd., Männedorf, Switzerland). MIC changes of four-fold or greater were considered significant. The combination of gentamicin and phenylalanine-arginine β-naphthylamide (PAβN) (Sigma-Aldrich Inc., St.Louis, MO, USA) at a concentration of 25 μg/mL was used as a positive control. Ultrapure water and a sterile NB medium were used as negative controls and had no effect on bacterial growth. The tested extracts could be considered as EPIs if they reduced the gentamicin MIC by at least four-fold.

## 5. Conclusions

Our new data provide significant insights into the chemical composition and antibacterial efficacy (MIC and efflux pump inhibitory activity) of six *S. maltophilia* strains of aqueous and DCM fractions extracted from *P. vittata* L. and *F. japonica* plants native to the northern Vietnam. The *F. japonica* DCM fraction, with emodin, emodin dianthrone, physcion, torosachrysone, and fallopion isomers as major components, exhibited the most significant activity, reducing the MIC of gentamicin four-fold for the BurA1 strain, eight-fold for the BurE1 strain at 250 μg/mL, and four-fold for the BurE1 strain at 125 μg/mL. The separation, purification, and identification of potential herbal-derived compounds with efflux pump inhibitory activities will undoubtedly foster a better understanding of the application of EPIs when combined with antibiotics for the treatment of (multi)drug-resistant bacterial infections.

## Figures and Tables

**Table 1:** Chemical composition of aqueous and dichloromethane (DCM) phases of *Pteris vittata* L. and *Fallopia japonica*.

	*P. vittata* L.	*F. japonica*
Aqueous phase	Procyanidin trimer A-type ^a^ Procyanidin dimer digallate A-type ^a^ Procyanidin tetramer A-type ^a^ 4- caffeoylquinic acid 3- caffeoylquinicacid p-coumaroyl pentoside acid	*trans*-Piceid and its isomer ^a^ *trans*-Resveratrol ^a^ Oxyresveratrolglucoside ^a^ Proanthocyanidin tetramer B-type Proanthocyanidin trimer B-type Proanthocyanidin dimer B-type Taxifolin
DCM phase	Unidentified diterpene ^a^	Emodin ^a^ Emodin dianthrone and its isomer ^a^ Torosachrysone ^a^ Physcion ^a^ Fallopion and its isomer ^a^ Resveratrol derivative

^a^ Major constituent.

**Table 2:** Antibacterial activities (Minimum inhibitory concentration (MIC) expressed in μg/mL) of *P. vittata* L. and *F. japonica* aqueous and DCM fractions on six *S. maltophilia* strains.

Samples		Clinical Strains			Environmental Strains		
Plant	Solvent	502	0366	K279a	PierC1	BurA1	BurE1
*P. vittata* L.	H_2_O	NA *	>2000	>2000	>2000	>2000	>2000
	DCM	>2000	>2000	>2000	>2000	>2000	>2000
*F. japonica*	H_2_O	>2000	>2000	>2000	>2000	>2000	>2000
	DCM	>1000	31.25	>1000	NA	>1000	NA

* NA: The concentration at which the extract exhibited the highest antibacterial activity was determined, but inhibition level was less than 90% of bacterial growth in the negative control.

**Table 3:** MICs (μg/mL) of gentamicin (GEN) (the concentrations range from 2.5 to 320 μg/mL) in the absence and presence of an efflux pump inhibitor (EPI), phenylalanine-arginine β-naphthylamide (PAβN), at concentration of 25 μg/mL. The test was performed on six *S. maltophillia* strains.

*S. maltophillia* Strains	502	0366	K279a	PierC1	BurA1	BurE1
GEN	20	5	80	10	40	320
GEN+PAβN	>80	20	40	10	20	80

**Table 4:** Reduction of gentamicin’s MICs in presence of four extracts of *P. vittata* L. and *F. japonica*.

Concentration (μg/mL)	*P. vittata* L.		*F. japonica*	
	H_2_O	DCM	H_2_O	DCM
250	(X)	2-fold for BurE1	(X)	4-fold for BurA1 8-fold for BurE1
125				4-fold for K279a, BurA1 4-fold for BurE1
62.5				2-fold for K279a, BurE1

(X) extract did not cause MIC to change.

**Table 5:** Levels by which emodin, physcion, sennidine A, and sennidine B (30 μg/mL) inhibited bacterial growth, expressed as a percentage (%) and based on bacterial growth in the negative control (dimethyl sulfoxide, 0.5%).

	K279a	BurA1	BurE1
Emodin	6.28 ± 0.20	−2.05 ± 1.47	1.37 ± 2.06
Physcion	−1.75 ± 0.88	−1.22 ± 0.70	−1.34 ± 1.75
Sennidine A	−8.56 ± 0.95	2.07 ± 0.75	1.50 ± 0.28
Sennidine B	−7.88 ± 2.05	4.02 ± 1.41	0.81 ± 0.51

## Data Availability

Not applicable.

## References

[1] Gil-Gil T., Martínez J.L., Blanco P. (2020). Mechanisms of antimicrobial resistance in *Stenotrophomonas maltophilia*: A review of current knowledge. Expert Rev. Anti Infect. Ther..

[2] Youenou B., Favre-Bonté S., Bodilis J., Brothier E., Dubost A., Muller D., Nazaret S. (2015). Comparative Genomics of Environmental and Clinical *Stenotrophomonas maltophilia* Strains with Different Antibiotic Resistance Profiles. Genome Biol. Evol..

[3] Henderson P.J.F., Maher C., Elbourne L.D.H., Eijkelkamp B.A., Paulsen I.T., Hassan K.A. (2021). Physiological Functions of Bacterial “Multidrug” Efflux Pumps. Chem. Rev..

[4] Tegos G.P., Haynes M., Strouse J.J., Khan M.M.T., Bologa C.G., Oprea T.I., Sklar L.A. (2011). Microbial efflux pump inhibition: Tactics and strategies. Curr. Pharm. Des..

[5] Michalet S., Rouifed S., Pellassa-Simon T., Fusade-Boyer M., Meiffren G., Nazaret S., Piola F. (2017). Tolerance of Japanese knotweed s.l. to soil artificial polymetallic pollution: Early metabolic responses and performance during vegetative multiplication. Environ. Sci. Pollut. Res..

[6] Pham H.N., Michalet S., Bodillis J., Nguyen T.D., Nguyen T.K.O., Le T.P.Q., Haddad M., Nazaret S., Dijoux-Franca M.-G. (2017). Impact of metal stress on the production of secondary metabolites in *Pteris vittata* L. and associated rhizosphere bacterial communities. Environ. Sci. Pollut. Res. Int..

[7] Zhang Y., Zheng L., Zheng Y., Xue S., Zhang J., Huang P., Zhao Y., Hao X., He Z., Hu Z. (2020). Insight into the assembly of root-associated microbiome in the medicinal plant *Polygonum cuspidatum*. Ind. Crops Prod..

[8] Ma L.Q., Komar K.M., Tu C., Zhang W., Cai Y., Kennelley E.D. (2001). A fern that hyperaccumulates arsenic. Nature.

[9] An Z.-Z., Huang Z.-C., Lei M., Liao X.-Y., Zheng Y.-M., Chen T.-B. (2006). Zinc tolerance and accumulation in *Pteris vittata* L. and its potential for phytoremediation of Zn- and As-contaminated soil. Chemosphere.

[10] Danh L.T., Truong P., Mammucari R., Foster N. (2014). A critical review of the arsenic uptake mechanisms and phytoremediation potential of *Pteris vittata*. Int. J. Phytoremediation.

[11] Anh B.T.K., Kim D.D., Tua T.V., Kien N.T., Anh D.T. (2011). Phytoremediation potential of indigenous plants from Thai Nguyen province, Vietnam. J. Environ. Biol. Acad. Environ. Biol. India.

[12] Bui T.K.A., Dang D.K., Nguyen T.K., Nguyen N.M., Nguyen Q.T., Nguyen H.C. (2014). Phytoremediation of heavy metal polluted soil and water in Vietnam. J. Vietnam. Environ..

[13] Nguyen N.-L., Bui V.-H., Pham H.-N., To H.-M., Dijoux-Franca M.-G., Vu C.-T., Nguyen K.-O.T. (2022). Ionomics and metabolomics analysis reveal the molecular mechanism of metal tolerance of *Pteris vittata* L. dominating in a mining site in Thai Nguyen province, Vietnam. Environ. Sci. Pollut. Res..

[14] Nguyen Thi K.-O., Nguyen N.-L., Pham H.-N., Sawada Y., Hirai M.Y., Dauwe R., Dijoux-Franca M.-G. (2021). Development of a *Pteris vittata* L. compound database by widely targeted metabolomics profiling. Biomed. Chromatogr..

[15] Paul T., Das B., Apte K. (2012). Suchitra Hypoglycemic Activity of *Pteris vittata* L., a Fern on Alloxan Induced Diabetic Rats. Inven. Rapid Planta Act..

[16] Gong X.-L., Chen Z.-H., Liang N.-C. (2007). Advances in study on chemical constituents and pharmacological activities of plants of genus *Pteris*. Zhongguo Zhong Yao Za Zhi Zhongguo Zhongyao Zazhi China Chin. Mater. Medica.

[17] Dupuy J., Larrieu G., Sutra J.F., Lespine A., Alvinerie M. (2003). Enhancement of moxidectin bioavailability in lamb by a natural flavonoid: Quercetin. Vet. Parasitol..

[18] Choi J.-S., Jo B.-W., Kim Y.-C. (2004). Enhanced paclitaxel bioavailability after oral administration of paclitaxel or prodrug to rats pretreated with quercetin. Eur. J. Pharm. Biopharm. Off. J. Arb. Pharm. Verfahr. EV.

[19] Wang Y.-H., Chao P.-D.L., Hsiu S.L., Wen K.-C., Hou Y.-C. (2004). Lethal quercetin-digoxin interaction in pigs. Life Sci..

[20] Chiruvella K.K., Mohammed A., Dampuri G., Ghanta R.G., Raghavan S.C. (2007). Phytochemical and Antimicrobial Studies of Methyl Angolensate and Luteolin-7-O-glucoside Isolated from Callus Cultures of *Soymida febrifuga*. Int. J. Biomed. Sci. IJBS.

[21] Le H.-L., To D.-C., Tran M.-H., Do T.-T., Nguyen P.-H. (2020). Natural PTP1B Inhibitors From *Polygonum cuspidatum* and Their 2-NBDG Uptake Stimulation. Nat. Prod. Commun..

[22] Zhang H., Li C., Kwok S.-T., Zhang Q.-W., Chan S.-W. (2013). A Review of the Pharmacological Effects of the Dried Root of *Polygonum cuspidatum* (Hu Zhang) and Its Constituents. Evid. Based Complement. Alternat. Med..

[23] Lee C.-C., Chen Y.-T., Chiu C.-C., Liao W.-T., Liu Y.-C., David Wang H.-M. (2015). *Polygonum cuspidatum* extracts as bioactive antioxidaion, anti-tyrosinase, immune stimulation and anticancer agents. J. Biosci. Bioeng..

[24] Cucu A.-A., Baci G.-M., Dezsi Ş., Nap M.-E., Beteg F.I., Bonta V., Bobiş O., Caprio E., Dezmirean D.S. (2021). New Approaches on Japanese Knotweed (*Fallopia japonica*) Bioactive Compounds and Their Potential of Pharmacological and Beekeeping Activities: Challenges and Future Directions. Plants.

[25] Li L., Song X., Yin Z., Jia R., Li Z., Zhou X., Zou Y., Li L., Yin L., Yue G. (2016). The antibacterial activity and action mechanism of emodin from *Polygonum cuspidatum* against *Haemophilus parasuis* in vitro. Microbiol. Res..

[26] de Oliveira-Garcia D., Dall’Agnol M., Rosales M., Azzuz A.C.G.S., Alcántara N., Martinez M.B., Girón J.A. (2003). Fimbriae and adherence of *Stenotrophomonas maltophilia* to epithelial cells and to abiotic surfaces. Cell. Microbiol..

[27] Alonso A., Sanchez P., Martínez J.L. (2000). *Stenotrophomonas maltophilia* D457R contains a cluster of genes from gram-positive bacteria involved in antibiotic and heavy metal resistance. Antimicrob. Agents Chemother..

[28] Zhang L., Li X.Z., Poole K. (2001). Fluoroquinolone susceptibilities of efflux-mediated multidrug-resistant *Pseudomonas aeruginosa, Stenotrophomonas maltophilia* and *Burkholderia cepacia*. J. Antimicrob. Chemother..

[29] Li X., Hu J., Wang B., Sheng L., Liu Z., Yang S., Li Y. (2014). Inhibitory effects of herbal constituents on P-glycoprotein in vitro and in vivo: Herb-drug interactions mediated via P-gp. Toxicol. Appl. Pharmacol..

[30] Li X., Dong Y., Wang W., Wang H., Chen Y., Shi G., Yi J., Wang J. (2013). Emodin as an effective agent in targeting cancer stem-like side population cells of gallbladder carcinoma. Stem Cells Dev..

[31] Bhardwaj A.K., Mohanty P. (2012). Bacterial efflux pumps involved in multidrug resistance and their inhibitors: Rejuvinating the antimicrobial chemotherapy. Recent Patents Anti-Infect. Drug Disc..

[32] Rao M., Padyana S., Dipin K.M., Kumar S., Nayak B.B., Varela M.F. (2018). Antimicrobial Compounds of Plant Origin as Efflux Pump Inhibitors: New Avenues for Controlling Multidrug Resistant Pathogens. J. Antimicrob. Agents.

[33] Huang Y.-Q., Huang G.-R., Wu M.-H., Tang H.-Y., Huang Z.-S., Zhou X.-H., Yu W.-Q., Su J.-W., Mo X.-Q., Chen B.-P. (2015). Inhibitory effects of emodin, baicalin, schizandrin and berberine on *hefA* gene: Treatment of *Helicobacter pylori*-induced multidrug resistance. World J. Gastroenterol. WJG.

[34] Lechner D., Gibbons S., Bucar F. (2008). Plant phenolic compounds as ethidium bromide efflux inhibitors in *Mycobacterium smegmatis*. J. Antimicrob. Chemother..

[35] Saha P., Rahman F.I., Hussain F., Rahman S.M.A., Rahman M.M. (2022). Antimicrobial Diterpenes: Recent Development From Natural Sources. Front. Pharmacol..

[36] Singh H. (1999). Potential medicinal pteridophytes of India and their chemical constituents. J. Econ. Tax. Bot..

[37] Nath K., Bhattacharya M.K., Kar S. (2018). Antimicrobial Potential of Ethnomedicinal Ferns of Southern Assam, India. Indian J. Pharm. Sci..

[38] Gibb J., Wong D.W. (2021). Antimicrobial Treatment Strategies for *Stenotrophomonas maltophilia*: A Focus on Novel Therapies. Antibiotics.

[39] Zając O.M., Tyski S., Laudy A.E. (2022). Phenotypic and Molecular Characteristics of the MDR Efflux Pump Gene-Carrying *Stenotrophomonas maltophilia* Strains Isolated in Warsaw, Poland. Biology.

[40] Zhang L., Li X.-Z., Poole K. (2001). SmeDEF Multidrug Efflux Pump Contributes to Intrinsic Multidrug Resistance in *Stenotrophomonas maltophilia*. Antimicrob. Agents Chemother..

[41] Gould V.C., Okazaki A., Avison M.B. (2013). Coordinate Hyperproduction of SmeZ and SmeJK Efflux Pumps Extends Drug Resistance in *Stenotrophomonas maltophilia*. Antimicrob. Agents Chemother..

[42] Impey R.E., Hawkins D.A., Sutton J.M., Soares da Costa T.P. (2020). Overcoming Intrinsic and Acquired Resistance Mechanisms Associated with the Cell Wall of Gram-Negative Bacteria. Antibiot. Basel Switz..

[43] Sun J., Deng Z., Yan A. (2014). Bacterial multidrug efflux pumps: Mechanisms, physiology and pharmacological exploitations. Biochem. Biophys. Res. Commun..

[44] Huang Y.-W., Liou R.-S., Lin Y.-T., Huang H.-H., Yang T.-C. (2014). A Linkage between SmeIJK Efflux Pump, Cell Envelope Integrity, and σE-Mediated Envelope Stress Response in *Stenotrophomonas maltophilia*. PLoS ONE.

[45] Lin Y.-T., Huang Y.-W., Chen S.-J., Chang C.-W., Yang T.-C. (2015). The SmeYZ Efflux Pump of *Stenotrophomonas maltophilia* Contributes to Drug Resistance, Virulence-Related Characteristics, and Virulence in Mice. Antimicrob. Agents Chemother..

[46] Lin C.-W., Huang Y.-W., Hu R.-M., Yang T.-C. (2014). SmeOP-TolCSm Efflux Pump Contributes to the Multidrug Resistance of *Stenotrophomonas maltophilia*. Antimicrob. Agents Chemother..

[47] Lin Y.-T., Huang Y.-W., Liou R.-S., Chang Y.-C., Yang T.-C. (2014). MacABCsm, an ABC-type tripartite efflux pump of *Stenotrophomonas maltophilia* involved in drug resistance, oxidative and envelope stress tolerances and biofilm formation. J. Antimicrob. Chemother..

